# EZH2 Mediates Proliferation, Migration, and Invasion Promoted by Estradiol in Human Glioblastoma Cells

**DOI:** 10.3389/fendo.2022.703733

**Published:** 2022-02-07

**Authors:** Aylin Del Moral-Morales, Juan Carlos González-Orozco, Ana María Hernández-Vega, Karina Hernández-Ortega, Karla Mariana Peña-Gutiérrez, Ignacio Camacho-Arroyo

**Affiliations:** ^1^ Unidad de Investigación en Reproducción Humana, Instituto Nacional de Perinatología-Facultad de Química, Universidad Nacional Autónoma de México (UNAM), Ciudad de México, Mexico; ^2^ Departamento de Biología, Facultad de Química, Universidad Nacional Autónoma de México (UNAM), Ciudad de México, Mexico

**Keywords:** glioblastoma, 17β-estradiol, EZH2, estrogen receptor α, estrogen receptor β, PRC2

## Abstract

Glioblastomas (GBM) are the most frequent and aggressive brain tumors. 17β-estradiol (E2) increases proliferation, migration, and invasion of human GBM cells; however underlying mechanisms are no fully understood. Zeste 2 Enhancer Homologous enzyme (EZH2) is a methyltransferase part of Polycomb 2 repressor complex (PRC2). In GBM, EZH2 is overexpressed and involved in the cell cycle, migration, and invasion processes. We studied the role of EZH2 in the pro-oncogenic actions of E2 in human GBM cells. EZH2 gene silencing and pharmacological inhibition of EZH2 blocked proliferation, migration, and invasion of GBM cells induced by E2. We identified in silico additional putative estrogen response elements (EREs) at the EZH2 promoter, but E2 did not modify EZH2 expression. In silico analysis also revealed that among human GBM samples, EZH2 expression was homogeneous; in contrast, the heterogeneous expression of estrogen receptors (ERs) allowed the classification of the samples into groups. Even in the GBM cluster with high expression of ERs and those of their target genes, the expression of PCR2 target genes did not change. Overall, our data suggest that in GBM cells, pro-oncogenic actions of E2 are mediated by EZH2, without changes in EZH2 expression and by mechanisms that appear to be unrelated to the transcriptional activity of ERs.

## Introduction

Astrocytomas are primary tumors generated by the malignization of glial cells, glial progenitors, or transformed neural stem cells. According to their histopathological characteristics, the WHO classifies gliomas into four grades, grade IV or glioblastoma (GBM), as the most aggressive and frequent (1). GBMs are characterized by being highly invasive and fast-growing so that patients have a low life expectancy (12 to 16 months after diagnosis). Unfortunately, currently available therapies (surgery, chemotherapy, and radiation therapy) are not enough, as a 5-year survival rate is less than 5% (2, 3). Because GBMs are more frequent in males than in females, the role of sex hormones such as progesterone, 17β-estradiol (E2), and testosterone in the incidence and progression of GBM has gained greater attention (4–6). Notably, E2 concentration is higher in GBM biopsies than in low-grade gliomas (7).

Moreover, E2 promotes the proliferation, migration, and invasion of cells derived from human GBM (4, 8). It has been recently shown that E2 is essential for epithelial to mesenchymal transition (EMT) of human GBM cells (9), which suggests that this hormone and its mechanisms of action are of great interest for the study of GBMs. Estrogens bind to specific intracellular and membrane receptors in cells. There are two estrogen-specific intracellular receptor subtypes, estrogen receptors α and β (ERα and ERβ), which acts as ligand-activated transcription factors to regulate gene expression. Moreover, these receptors can associate with the plasma membrane, triggering intracellular signaling pathways (10, 11).

The Enhancer of Zeste Homolog 2 (EZH2) is an enzymatic part of the Polycomb Group (PcG), a set of transcriptional repressors that modify chromatin that controls the progression of the cell cycle and participates in the maintenance of cell differentiation (12). This group is represented by the Polycomb 1 and 2 repressor complexes (PRC1 and PRC2). PRC2 is the complex responsible for mono, di, and trimethylation of histone 3 in lysine 27 (H3K27me1/2/3) (13). The H3K27me3 tag is associated with gene promoters found in facultative heterochromatin and is read by PRC1, which binds to chromatin, preventing transcription (14). PRC2 has three essential core components: EZH2, a lysine-specific methyltransferase presenting a SET [Su(var) 3-9; E(z); Trithorax] catalytic domain; EED 1 (embryonic ectoderm development) containing WD40 [tryptophan–aspartate (WD) repeat] motifs that identify adjacent methylation marks in histones and anchor the complex to chromatin; and SUZ12, (Suppressor of Zeste 12) [Su(z)12 or its human homolog, JJAZ1], a histone deacetylase (15–17). EZH2 is known to be required to maintain cell identity and differentiation by repressing tissue-specific genes and proliferation inhibitors (12).

EZH2 is dysregulated in various types of cancer, including brain tumors (18–20). In GBMs, EZH2 functions as an oncogene. It is involved in multiple glioma cellular processes, including cell cycle, invasion, and glioma stem cell maintenance, which is thought to be responsible for drug resistance and tumor recurrence (13, 21). Besides, the expression of this gene is directly related to tumor malignancy, and its overexpression is associated with poor prognosis (19–21). Unfortunately, not much is known about the factors involved in regulating EZH2 gene expression and activity in GBM. However, in breast cancer, estrogen response elements (EREs) have been reported in promoter sequences of EZH2; moreover, E2 directly regulates the expression of EZH2 through its nuclear receptors (22). Also, in papillary thyroid carcinoma, E2 interacted with ERα and upregulated EZH2 (23). Given the above evidence, we speculate that in GBM, EZH2 expression could be regulated by E2 and that EZH2 participates in estrogenic actions on proliferation, migration, and invasion of GBM cells.

The present study characterized the EZH2 expression in human GBM-derived cell lines (U87, U251, and D54) and a set of glioma biopsies data from The Cancer Genome Atlas (TCGA). E2 induced no significant changes in EZH2 expression in the cell lines. However, silencing EZH2 or inhibiting its activity suppressed E2-induced proliferation, migration and invasion in GBM cells. Interestingly, in silico data analysis of GBM biopsies showed that ERα and ERβ were heterogeneously expressed, so we grouped them into three hierarchical clusters. GBM cluster 3, with the highest expression of ERs and enriched in estrogen-regulated genes, showed no changes in PRC2/EZH2 target genes. Therefore, our results indicate that pro-oncogenic actions of E2 on GBM cells are mediated *via* EZH2 activity, without E2 modifying EZH2 expression. In GBM samples, the expression of ERs and their transcriptional actions were not directly related to those of EZH2, suggesting that E2 should modulate EZH2 by an extra-nuclear mechanism.

## Materials and Methods

### Cell Culture and Treatments

Human GBM-derived cell lines U87, U251, and D54 (ATCC, WA, USA) were cultured in Dulbecco’s Modified Eagle’s medium (DMEM, *In vitro*, Mexico) high glucose supplemented with 10% fetal bovine serum (FBS), 1 mM pyruvate, 2 mM glutamine, and 0.1 mM non-essential amino acids. Cell cultures were maintained at 37°C in a humidified atmosphere with 5%. 24 h before treatments, the medium was changed by phenol red-free DMEM (*In vitro*, Mexico) supplemented with charcoal-stripped FBS (HyClone, USA). The following treatments were applied for 12 or 24 h: E2 (1 nM, 10 nM, 100 nM, and 1 μM) and vehicle (V, 0.02% cyclodextrin). Cells were also treated with 5 µM of GSK 343 (an S-adenosyl methionine, SAM-competitive inhibitor) or vehicle control (0.1% DMSO) to evaluate the effect of pharmacological inhibition of EZH2 (24). E2, Cyclodextrin (CDX), GSK 343 and DMSO were purchased from Sigma Aldrich (USA).

### RT-qPCR

According to the manufacturer’s protocol, total RNA was extracted using TRIzol LS Reagent (Thermo Fisher Scientific, USA), and concentration was measured by spectrophotometry (NanoDrop 2000 Spectrophotometer, Thermo Fisher Scientific, USA). RNA integrity was verified by electrophoresis with a 1.5% agarose gel in Tris-Borate-ethylenediaminetetraacetic acid (EDTA) buffer using GreenSafe for visualization. Total RNA from Healthy Human Astrocytes (HA) was obtained from ScienCell Research Laboratories (USA). Total RNA (1µg) was subjected to reverse transcription using the MMLV RT enzyme (Invitrogen, USA) and oligo-dT12-18 primers. Complementary DNA (cDNA) was amplified by RT-qPCR using the FastStart DNA Master SYBR Green I reagent kit for LightCycler 1.5 (Roche Diagnostics, Germany) following the manufacturer’s protocol. Primers used were the following: EZH2 (25), (FW-5’-CCCTGACCTCTGTCTTACTTGTGGA-3’, RV-ACGTCAGATGGTGCCAGCAATA-3’; 18S (FW-5’AGTGAAACTGCAATGGCTC-3’, RV-5’-CTGACCGGGTTGGTTTTGAT-3’). Relative expression of the EZH2 gene was calculated considering the 18S mRNA gene as an endogenous reference. Relative expression levels were calculated by the 2^ΔΔCt^ method (26, 27).

### Western Blot

After treatments, cell pellets were lysed with RIPA buffer plus supplemented with protease inhibitors (1 mM EDTA, 2 µg/ml leupeptin, 2 µg/ml aprotinin, 1 mM PMSF). Total protein was obtained by centrifugation at 14,000 rpm, at 4°C for 5 min and quantified using Pierce Protein Assay reagent (Thermo Fisher Scientific, USA) in a NanoDrop 2000 Spectrophotometer (Thermo Scientific, USA). Thirty μg of total protein were separated on 10% SDS-polyacrylamide gel electrophoresis (PAGE) and transferred to a PVDF membrane at 25V in semi-dry conditions for 45 min. Membranes were blocked overnight with 5% bovine serum albumin (BSA, *In Vitro*, Mexico), at 4°C and then incubated for 24h with one of the following antibodies: EZH2 (5246S, Cell Signaling, USA), H3K27me3 (9733, Cell signaling, USA) and α-Tubulin (sc-398103, Santa Cruz Biotechnology, USA). Afterwards, membranes were incubated with horseradish peroxidase-conjugated secondary anti-rabbit (sc-2004, Santa Cruz Biotechnology, USA) at room temperature for 45 min. Finally, membranes were incubated with Super Signal West Femto Maximum Sensitivity Substrate (Thermo Scientific, USA) and then exposed to Kodak Biomax Light Film (Sigma-Aldrich, USA) for detecting immune complexes. Densitometric analysis of western blot bands was conducted using Image J 1.45S software (National Institutes of Health, USA). EZH2 and H3K27me3 contents were normalized to that of α-tubulin.

### Immunofluorescence

U87, U251, and D54 cells were fixed with 4% paraformaldehyde solution (PFA) at room temperature for 20 min and washed with PBS. Then, cells were permeabilized and blocked (1% BSA, 0.2% Triton X-100 in PBS) at room temperature for 30 min. Next, cells were incubated for 24 h with rabbit anti-EZH2 (5246S, Cell Signaling, USA) at 4°C and rinsed with PBST (PBS with 0.05% Tween). Later, cells were incubated with Goat anti-Rabbit IgG Alexa Fluor 647 (21246, Santa Cruz Biotechnology, USA) at room temperature for 60 min and rinsed with PBST. Nuclei were stained with 1 mg/mL Hoechst 33342 solution (Thermo Fisher Scientific, USA) at room temperature and again rinsed with PBST. Finally, cells were coverslipped using a fluorescence mounting medium (Polysciences Inc., USA) and visualized in an Olympus Bx43 microscope. For each condition, six arbitrary fields at 400X magnification were captured, and fluorescence density was measured with ImageJ software.

### Bioinformatic Analysis of Response Elements

Gene sequence was obtained from the Human Genome Resources at NCBI (https://www.ncbi.nlm.nih.gov/genome/gdv/browser/?context=genome&acc=GCF_000001405.38). The promoter regions and transcription start site (TSS) were determined through the Ensembl database (28). Putative binding sites for ERs were searched with JASPAR (29), HOCOMOCO v.11 (30), and HOMER (v4.11) (31) platforms. Predicted binding sites by two or more databases with a score of 9 or greater and p-value < 0.05 were established as potential EREs.

### siRNA Transfection

siRNA-mediated silencing of EZH2 was used to evaluate whether E2 effects on GBM proliferation, migration, and cell invasion are mediated through EZH2. Briefly, 2.5×10^5^ U251 cells were seeded in 6-well plates in DMEM medium supplemented with 10% FBS and antibiotics. 24 h later, the medium was changed to DMEM phenol red-free medium without FBS and antibiotics. Then, using Lipofectamine RNAiMAX (Thermo Scientific, USA), cells were transfected with an EZH2 siRNA (10 nM, sc-35312, Santa Cruz Biotechnology, USA) or with a control siRNA (10 nM, sc-37007, Santa Cruz Biotechnology, USA) that does not induce any specific mRNA degradation. 24 h after siRNA transfection, the cell medium was refreshed, and 24 h later, cells were harvested for total protein extraction to determine EZH2 silencing efficiency through Western Blot.

### Cell Proliferation Assays

Briefly, 2.5×10^5^ U251 cells were transfected, as was described in the above section. 48 h after siRNA transfection, cells were treated with E2 (10 nM) or V (0.02% CDX) for 24 h. After treatment, cell proliferation was evaluated with the 5-Bromo-2’deoxyuridine (BrdU) incorporation Kit I (Roche, USA) following the manufacturer’s instructions. Additionally, the Hoechst staining (Thermo Scientific, USA) was used to counterstain the cell nuclei. BrdU and Hoechst fluorescence signals were analyzed under an Olympus Bx43F (Japan) microscope. The number of BrdU-positive cells was analyzed with the Image J software (NIH, USA). BrdU-positive cell percentage was calculated considering the total number of cell nuclei stained with Hoechst. In the case of pharmacological inhibition of EZH2, cells were treated with E2 (10 nM), CDX (0.0 2%) plus GSK 343 (5 μM), or vehicle (DMSO 0.01%) for 48 h. After treatments, cell proliferation was measured as described above.

### Migration Assays

After siRNA transfection, cells were cultured for 24 h in phenol red-free DMEM medium supplemented with 10% hormone-free FBS. A scratch was made in the cell monolayer using a 200 μl pipette tip, the removed cells were rinsed with PBS, and DMEM supplemented with hormone-free SFB was refreshed. One hour before adding treatments, cells were incubated with β-D-arabinofuranoside (Ara-C, 10 µM, Sigma-Aldrich, USA) to inhibit cell proliferation; then, cells were treated with E2 (10 nM) or V (0.02% CDX) for 24 h. To assess migration under conditions of inhibition of EZH2 activity, cells were treated with E2 (10 nM), CDX (0.0 2%) plus GSK 343 (5 μM), or vehicle (DMSO 0.01%) for 24 h. Images 100X of the wound area were captured at 0, and 24 h post-treatment with an Infinity 1-2 camera (Lumenera, Canada) attached to an Olympus CKX41 inverted microscope. Relative migration area distance (%) was calculated in four random fields of each experimental condition using the MRI Wound Healing Tool plugins of Image J software (National Institute of Health, USA).

### Viability Assays

2.5×10^5^ U251 cells were grown in DMEM medium supplemented with 10% FBS and antibiotics. 24 h later, the medium was changed to DMEM phenol red-free medium without FBS and antibiotics. Then, cells were transfected with an EZH2 siRNA (10153 nM, sc-35312, Santa Cruz Biotechnology, USA) or with a control siRNA (10 nM, sc-37007, Santa Cruz Biotechnology, USA). 48 h after siRNA transfection, cells were treated with E2 (10 nM) or V (0.02% CDX) for 24 h and then harvested with 1mL PBS-EDTA (1mM) and stained with trypan blue (0.4%). Viable cells were quantified with Countess II cell counter (Thermo Fisher Scientific, MA, USA).

### Invasion Assays

The invasion potential of the cells was tested through Transwell assay using 10 μm membrane thickness and 8 μm pore size Transwell inserts (3422, Corning, Corning, USA) in 24-well plates. Extracellular matrix (ECM) gel from Engelbreth-Holm-Swarm murine sarcoma (2 mg/ml, matrigel E1270 Sigma-Aldrich, USA) was diluted with phenol red-free DMEM medium without supplement. It was placed in each well (50 µl) and incubated at 37°C for 2 h. Next, on top of Transwell inserts, 2.5x10^5^ cells were incubated in non-supplemented, phenol red-free DMEM medium (150 µl) with Ara-C (10 µM), and treatments (10 nM E2 or V). In the case of EZH2 inhibition, GSK 343 (5 μM), or vehicle (DMSO 0.01%) were also used. The bottom part of the Transwell inserts was filled with 500 µl of phenol red-free DMEM supplemented with 10% hormone-free FBS, acting as a chemoattractant. Then, the plate was incubated for 24 h at 37°C. Finally, after incubation for 24 h, cells from the upper surface of the membrane Transwells were washed, and cells that penetrated in matrigel (invading cells) were fixed with PFA 4% for 20 min and then stained with 0.1% crystal violet for 20 min. Images of invading cells were acquired at 100X magnification with an Infinity 1-2C camera (Lumenera, Canada) connected to an inverted microscope (CKX41, Olympus, Japan). Cell number of four random fields per condition was determined with the Cell Counter plugin in the ImageJ software (National Institute of Health, USA).

### Analysis of RNA-seq Data From TCGA and GTEx

Ribonucleic acid sequencing (RNA-seq) data of primary tumors from the low-grade gliomas (LGG, n=196) and GBM (n=139) projects of The Cancer Genome Atlas (TCGA-LGG and TCGA-GBM) were downloaded from the Genomic Data Commons portal of the National Cancer Institute (USA, https://gdc.cancer.gov/) using the “TCGAbiolinks” package for R v.3.5 (32). The transcriptome of 249 healthy cerebral cortex tissue samples was obtained from the GTEx database (https://gtexportal.org/home/). LGG includes grade I, II, and III gliomas. Data normalization and differential expression analysis were carried out with the DESeq2 v.1.22.2 (33) package. The graphs were built with the ggplot2 v3.2.1 (34) package.

Gene set enrichment analysis (GSEA) was carried out with the software of the same name, GSEA v.4.01 (35). All the gene sets used are available on the GSEA website https://www.gsea-msigdb.org/gsea/index.jsp. Gene ontology analysis was performed on the Enrichr platform (https://maayanlab.cloud/Enrichr/).

### Statistical Analysis

Data from TCGA and GTEx were plotted and analyzed using R version 3.5.2. At least three biologicals replicates for each experiment approach were done. Experimental data were analyzed and plotted using the GraphPad Prism 5.0 software (GraphPad Software, CA, USA). Statistical analysis among groups was performed using a one-way ANOVA with a Tukey post-test. p<0.05 was considered statistically significant.

## Results

### EZH2 Is Expressed in Human GBM-Derived Cells

**Figure 1:**
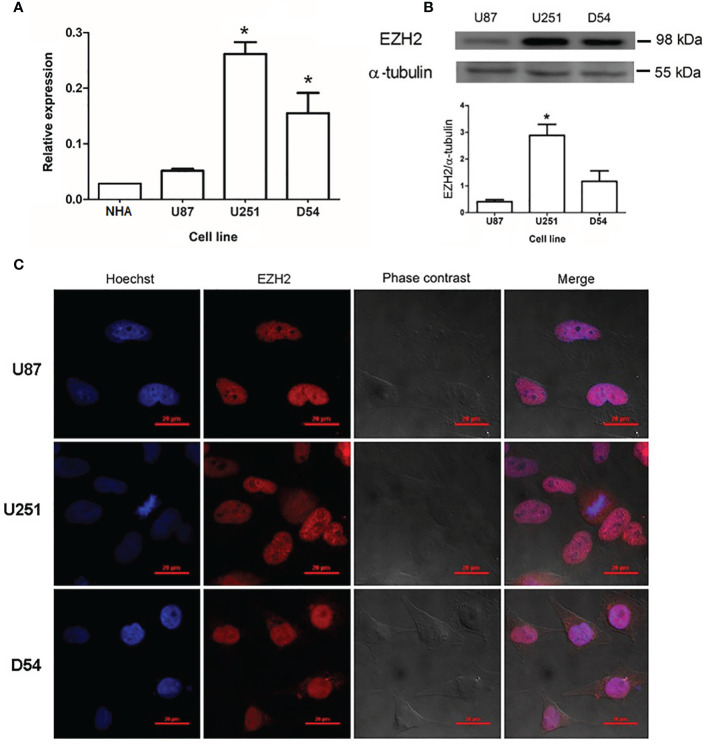
Expression of EZH2 at mRNA and protein levels in human GBM-derived cells. **(A)** RT-qPCR quantified the amount of EZH2 mRNA (normalized to 18S ribosomal RNA using the comparative 2^ΔΔCt^ method) in normal human astrocytes (NHA) and U87, U251, and D54 cell lines cultured under basal conditions. *p < 0.05 *vs*. U87 and NHA. Each bar represents the mean ± S.E.M., n = 3. **(B)** EZH2 content was determined by Western blot in U87, U251, and D54 cell line, using α-tubulin as a loading control. Representative blot image and the corresponding densitometric analysis for EZH2 content in human GBM-derived cells. *p < 0.05 *vs*. U87 and D54. Bars represent the mean ± S.E.M., n=3. **(C)** Subcellular localization of EZH2 in GBM cell lines. Representative images of immunofluorescence showed that EZH2 is mainly found in the nuclei. Images were captured at 100X amplification. Red scale bars = 20 μm.

The expression of EZH2 was analyzed in three different cell lines derived from human GBM (U87, U251, and D54) and compared to those of normal human astrocytes (NHA) (**Figure 1A**). All three human GBM-derived cell lines (U87, U251, and D54) expressed EZH2 under basal conditions at the mRNA level. EZH2 expression in U251 and D54 was significantly higher than that of NHA. Significant differences were also found among cell lines, with U251 showing the highest expression level and U87 the lowest. The protein levels of EZH2 were also evaluated through Western blot in the GBM cell lines, and consistent with mRNA analysis, U251 cells showed the highest EZH2 content, while U87 cells had the lowest content (**Figure 1B**).

Subcellular localization of EZH2 was analyzed by immunofluorescence in U87, U251, and D54 cell lines. EZH2 was expressed in all cell lines, and it was mainly found in the nucleus, where it colocalizes with Hoechst dye, as shown in **Figure 1C**. As a negative control, immunofluorescence was performed without the monoclonal antibody against EZH2 (**Supplementary Figure 1**).

### E2 Does Not Regulate EZH2 Expression in Human GBM

**Figure 2:**
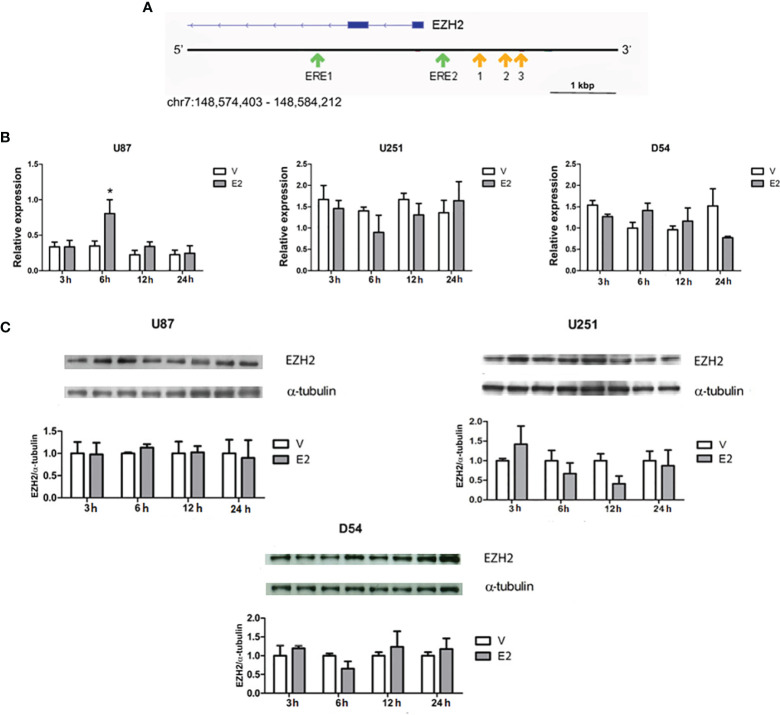
E2 effect on EZH2 expression. **(A)** Schematic representation of the EZH2 gene is depicted in blue. The locations of EREs predicted by an *in silico* analysis are marked with green arrows. The analysis was performed on three different platforms (JASPAR, HOCOMOCO, and HOMER). A potential binding site was considered those predicted by two or more databases with a score value of 9 or higher and p < 0.05. Yellow arrows point to the binding sites previously described (22). Scale bar =1 Kbp. **(B)** EZH2 expression was quantified by RT-qPCR after cell treatments with E2 (10 nM) or V (0.02% CDX) for 3, 6, 12, and 24 h. Relative expression of EZH2 mRNA was calculated by the 2^ΔΔCt^ method using 18S ribosomal RNA as a reference gene. *p < 0.001 vs. V. Results are presented as mean ± S.E.M. n = 3. **(C)** E2 effect on EZH2 content was also evaluated by Western blot. Densitometric analysis and corresponding representative images of EZH2 bands are shown. Each bar represents the mean ± S.E.M, n = 4.

EREs have been reported in the EZH2 promoter, showing that E2 regulates the expression of the EZH2 gene in a couple of cancer cell types (22, 23). In addition to the EREs previously described (22), we found two potential EREs in the promoter region of the EZH2 gene by an *in silico* analysis (**Figure 2A**). The sequences of these putative binding sites and those early validated are shown in **Table 1**.

**Table 1:** Sequences of EREs in EZH2 promoter.

Element	Position	Strand	Sequence
ERE1	-354 to -368	–	ATGTCTCCCGGTCCC
ERE2	+1599 to + 1586	–	TAATAACTTGCTTG
Sites reported by Bhan et al., 2016 (22).			
1	-846 to -859		GACCAGCCTGACC
2	-1238 to -1251	–	CGATCTCCTGACC
3	-1488 to -1501	–	AGGTAGCTTGACC

To evaluate the E2 regulation of EZH2 expression in GBM cells, U251, U87, and U251 cells were treated with E2 (10 nM) (4, 9) for 3, 6, 12, and 24 h. After treatment, EZH2 mRNA was quantified by RT-qPCR. Data showed that E2 significantly induced EZH2 expression in the U87 line at 6 h of treatment. However, no significant changes were observed in U251 or D54 cells (**Figure 2B**). A curve of E2 concentrations was performed to rule out the possibility of a dose-dependent effect. The expression of EZH2 was evaluated through RT-qPCR at 12 and 24 h after treatment in U251 cells. However, no significant changes were detected in EZH2 gene expression (**Supplementary Figure 2** **).** E2 effect on EZH2 protein content was also analyzed by Western blot. E2 induced no significant changes in any cell lines at any evaluated times (**Figure 2C**). Therefore, it suggests that E2 does not regulate the expression of EZH2 in cell lines U87, U251, and D54.

### EZH2 Silencing Inhibits E2-Induced Proliferation, Invasion, and Migration

**Figure 3:**
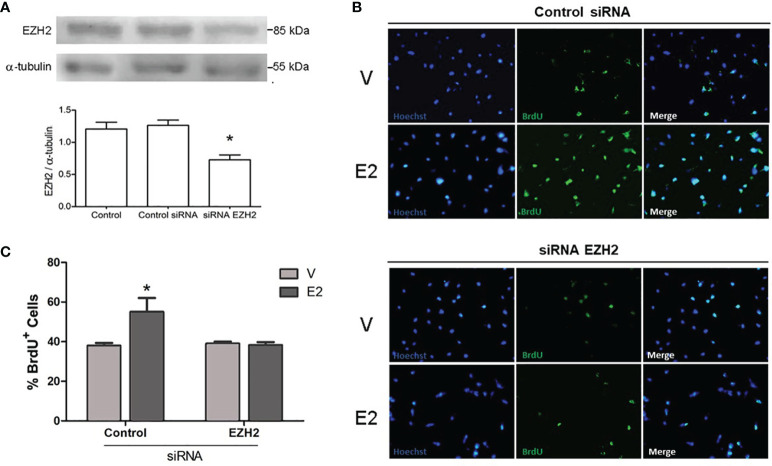
Effect of EZH2 silencing on E2-induced proliferation. **(A)** Validation of EZH2 silencing. Representative Western blots of EZH2 content in U251 cells transfected with EZH2 siRNA or control siRNA interference (aleatory RNA sequence). Control: cells treated only with transfection reagent. Densitometric data are shown as mean ± S.E.M. *p <0.05. vs. the other groups. Analysis of proliferative capacity of U251 cells transfected with control siRNA or EZH2 siRNA and treated with E2 (10 nM) or V (0.02% CDX) for 48 h. **(B)** Representative images of BrdU incorporation in proliferating U251 cells, **(C)** and its corresponding quantification graphs showing the percentage of BrdU positive cells. Bars represent the mean ± S.E.M. n =3. *p < 0.05. vs. the other groups.

In gliomas, EZH2 is involved in proliferation, invasion, and migration (19–21). E2 can also regulate these processes in GBM cells (4, 8, 9). Therefore, we decided to evaluate the impact of siRNA silencing of EZH2 on the pro-oncogenic actions E2-induced in the U251 cell line since it presented the highest levels of EZH2. First, the silencing efficiency of the siRNAs used was verified through Western blot. A decrease of close to 50% in EZH2 protein content was observed (**Figure 3A**). Also, we measured cell viability by trypan blue exclusion assay in U251 cells treated with E2 (10 nM) and CDX after EZH2 inhibition by siRNA. We found no significant changes between treatments (**Supplementary Table 1**).

To determine whether the silencing of EZH modifies the proliferation induced by E2 in U251 cells, we performed a BrdU incorporation assay. E2 significantly stimulated the proliferation of U251 cells transfected with the control siRNA, compared to vehicle. However, in cells with silenced EZH2, no significant differences were observed between E2 or vehicle treatments (**Figures 3B, C**). A significant reduction in proliferation was observed in cells treated with E2 and EZH2 siRNA than those treated with E2 and control siRNA. These results show that EZH2 mediates that proliferation induced by E2 in U251 cells.

**Figure 4:**
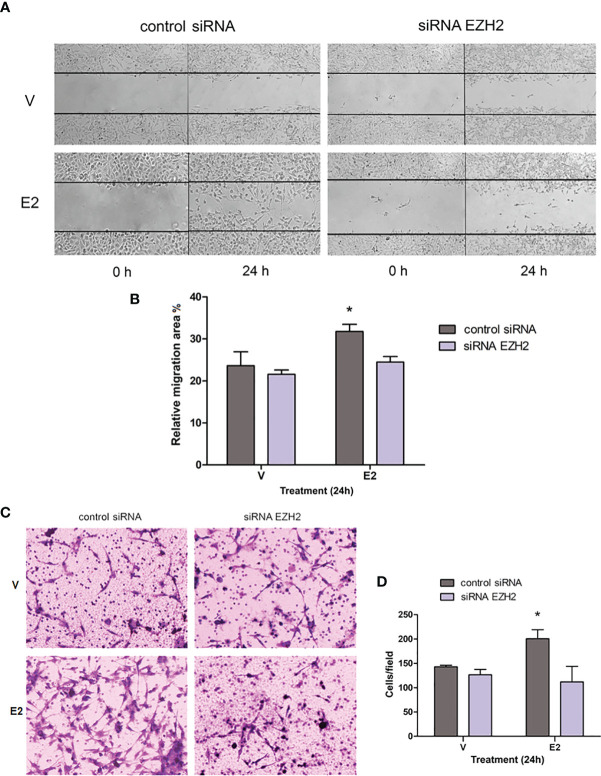
Effect of EZH2 silencing on E2-induced migration and invasion. **(A)** Wound healing assays were performed in the U251 cell line transfected with control siRNA or EZH2 siRNA and treated with E2 (10 nM) or V (0.02% CDX). Representative images of the scratch area at 0 and 24 h. **(B)** Relative migration area (%) of U251 cells into wound area was measured 24 h after scratch formation. Graph data are shown as mean ± S.E.M., n = 3. *p < 0.05. vs. the other groups. **(C)** Transwell assays were carried out in U251 cells treated as previously described. Representative photographs of the invading cells at the bottom of the transwells (cells that penetrated matrigel) stained with 0.1% cresyl violet after 24h of treatment **(D)** and the corresponding quantification. Results are expressed as the mean ± S.E.M., n = 3. *p < 0.05. vs. the other groups.

E2 also promotes migration and invasion of GBM-derived cell lines (4, 8, 9). To evaluate whether silencing of EZH2 also changes the migration induced by E2 in U251 cells, we carried out a wound-healing assay. Cells transfected with control siRNA and treated with E2 presented a higher migratory capacity than vehicle at 24 h after treatment. This increase was reversed when EZH2 was partially silenced (**Figures 4A, B**). A similar effect was observed when we evaluated invasiveness through a Transwell assay. E2 increased the number of invading cells (cells that penetrated in matrigel) transfected with control siRNA compared to the vehicle; however, in cells with silenced EZH2, we observed no significant differences in the cell number that penetrated the matrigel in E2 or vehicle conditions (**Figures 4C, D**). A significant diminution was found in invading cells treated with E2 and EZH2 siRNA compared with those treated with E2 and control siRNA. These observations show that EZH2 has an essential role in the promoting actions of E2 on proliferation, migration, and invasion in the U251 cell line.

### Inhibition of EZH2 Activity Suppresses E2-Induced Proliferation, Invasion, and Migration

Since EZH2 silencing inhibits the pro-oncogenic actions E2-induced in the U251 cell line, we decided to evaluate whether inhibition of EZH2 activity also suppresses E2-induced proliferation, migration, and invasion; we used GSK343, which functions as a SAM-competitive EZH2 inhibitor. Treatment with GSK 343 (5 µM) significantly reduced histone H3K27me3 in U251 cells, even when treated with E2 (**Supplementary Figure 3A**). Also, a substantial reduction in proliferation was observed in cells treated with E2 and GSK 343 than those treated with E2 and DMSO (**Supplementary Figures 3B, C**). These results further support EZH2 activity mediates proliferation induced by E2 in U251 cells.

Next, we performed wound-healing and transwell invasion assays after GSK 343 and E2 treatments. Similarly, GSK 343 (5 µM) suppressed the E2-induced increase in migratory (**Supplementary Figures 4A, B**) and invasive capacity (**Supplementary Figures 4C, D**) in U251 cells.

### Differential Expression of EZH2 and ERs in GBM Biopsies

The above observations indicate that EZH2 is essential for E2 pro-oncogenic actions, and since E2 could alternatively modulate EZH2 activity *via* nongenomic signaling involving ERs (ERα and ERβ) (36), we decided to explore whether there is a relationship between EZH2 and ERs expression in GBM. Therefore, transcriptomic data from low-grade glioma (LGG), GBM samples, and healthy human brain cortex samples obtained from the TCGA and GTEx platforms were analyzed.

Initially, we verified previous data indicating that EZH2 expression is higher in gliomas than in NT, increasing with tumor progression. EZH2 mRNA expression was higher in GBM than in LGG (**Supplementary Figure 5**), which supports the role of EZH2 in GBM progression (19–21). In an earlier report of our group, in silico analysis of a set of glioma samples revealed ERα expression was higher in NT than in gliomas. While, among gliomas, ERα mRNA was higher in GBM. Besides, a positive correlation of ER-β expression with astrocytoma malignancy progress was evidenced (9). These observations indicated that the expression of ERs mRNAs was heterogeneous and did not coincide with that observed in EZH2 mRNA (**Supplementary Figure 5**), suggesting that ERs and EZH2 expressions are not related in gliomas progression.

**Figure 5:**
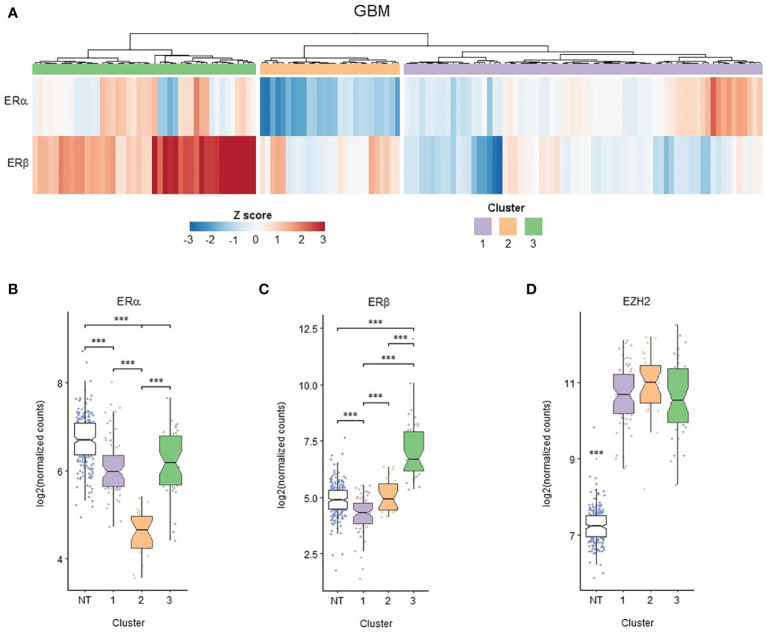
EZH2, ERα and ERβ expression clusters in human GBM biopsies. RNA-seq data obtained from TCGA. 139 primary GBM expression profiles of 249 healthy brain cortex samples (normal tissue, NT) obtained from the GTEx database were analyzed. **(A)** Heat map showing the expression of ERα and ERβ in each of the primary GBMs studied; based on it, three hierarchical clusters were defined. **(B)** ERα, **(C)** ERβ and **(D)** EZH2 expression in the different GBM groups formed and NT. ***p < 0.001. in the different sample groups.

To explore in detail the possibility of a relation between EZH2 and ERs expression, we focused our *in silico* analysis on GBM samples, which had the highest levels of EZH2. Since GBM samples presented a heterogeneous expression of ERα and ERβ, they were stratified using the hierarchical grouping method. Our analysis showed that three clusters of GBM samples are distinguishable, as shown in **Figure 5A**. In group 1, ERα expression was high, and ERβ expression was relatively low; group 2 had a slight expression of ERα, and medium expression of ERβ; and group 3 presented high levels of both receptors, ERα and ERβ (**Figures 5B, C**). The three GBM clusters had high EZH2 expression compared to NT; however, there were no significant differences (**Figure 5D**). In addition, we analyzed the sex composition of the different subgroups based on EZH2 and ERS expression, and we found that the composition of the GB subgroups is heterogeneous. There is no sexual dimorphism (**Supplementary Figures 6A**–**C**). Furthermore, the sex ratio is maintained in all three GB clusters, as indicated by a Chi-squared test (**Supplementary Figure 6D**).

We assessed whether the transcriptional activities of EZH2 and ERs are related to each other in GBM biopsy clusters with differential ERs expression levels. Differential gene expression data of GBM samples were used to carry out a Gene Set Enrichment Analysis (GSEA). In particular, we examined the behavior of two expression gene sets: early estrogenic response and proven targets of the PRC2 complex, to which EZH2 belongs. It was observed that group 3 had a higher enrichment of early estrogen-responsive genes than groups 1 and 2. However, there were no significant differences between clusters in terms of downregulation or upregulation of the target genes of PRC2 (**Supplementary Figure 7**).

**Figure 6:**
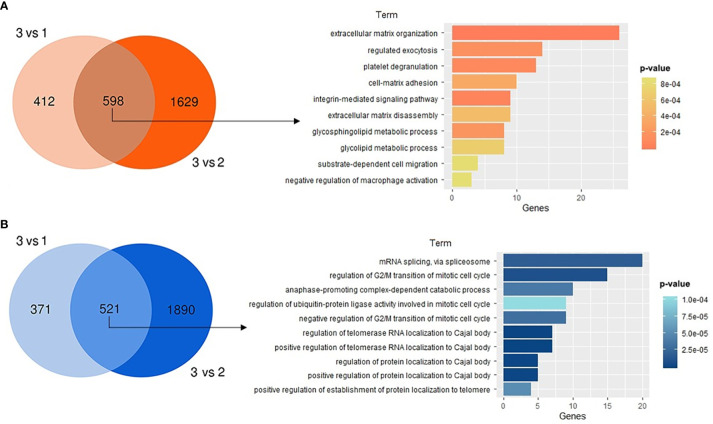
Gene ontology (GO) analysis for GBM group 3 regarding the other two GBM groups. Venn diagrams presenting the numerous differentially expressed genes and their corresponding top 10 enriched GO terms are shown for **(A)** upregulated or **(B)** downregulated regulated genes in cluster 3 vs clusters 1 and 2. In plots, for significant pathways listed, p-values are shown in colors.

Finally, to expand the scope of the principal pathways modulated in GBM group 3 regarding the other two GBM groups (groups 1 and 2), differential expression analysis and the corresponding gene ontology (GO) annotation were performed. A core of 598 upregulated and 521 downregulated genes in group 3 versus the other two groups were analyzed to identify the corresponding top 10 corresponding enriched processes (**Figures 6A, B**). The processes mainly enriched in the upregulated genes in cluster 3 are those involved in extracellular matrix (ECM) organization and disassembly, regulated exocytosis, platelet degranulation, cell-matrix adhesion, and integrin-mediated integration signaling, among others. Enriched terms in downregulated processes mainly included mRNA splicing by the spliceosome, mitotic cell cycle regulation processes and, localization control of telomerase RNA and proteins to Cajal bodies (CBs).

## Discussion

In this work, we evaluated the participation of the methyltransferase EZH2 on E2 mediated pro-oncogenic effects in GBM cells. Moreover, using in silico transcriptomic data of GBM samples, we analyzed if ERs activities, as transcriptional regulators, could be related to those EZH2, as an approach to explore whether E2 mediates EZH2 activation through a genomic mechanism *via* its ERs.

First, to characterize EZH2 expression in cell lines derived from human GBMs, baseline expression of the EZH2 gene was evaluated in U87, U251, and D54 cell lines and compared to that of healthy human astrocytes. It was found that the three lines differentially expressed EZH2 at basal conditions and to a higher level than the control tissue. In this regard, several reports indicate that EZH2 is overexpressed in biopsies from GBM patients and that its expression is related to the tumor grade (19–21). The inter-tumoral heterogeneity in GBM cell lines can reflect differences in proliferation rate and cell contact inhibition, even when grown under the same conditions (37). Additionally, immunofluorescence results indicated that EZH2 was mainly located in the nucleus, suggesting that EZH2 protein was in a functional state (by canonical mechanism) in the three GBM cell lines studied. In the case of U251 cells, we analyzed the content of H3K27me3, an indicator of EZH2 activity. Treatments with GSK 343, EZH2 inhibitor reduced histone H3K27trimethylation, suggesting that EZH2 present in U251 cells is active.

Considering that Bhan et al. previously reported the presence of EREs in the EZH2 promoter region (22) and that we found two others potential EREs through an *in silico* analysis, we decided to evaluate whether E2 (10 nM) treatments also regulate EZH2 expression in GBM cell lines. It is worth mentioning that the E2 (10 nM) concentration used is close to the E2 levels reported in GBMs biopsies (7). Moreover, it has been proven that E2 affects proliferation, migration, and invasion studies of GBMs cell lines (4, 8, 9). After E2 treatment, we observed no significant changes in EZH2 at either RNA or protein levels in GBM cells at any of the tested times. Even more, treatments with different concentrations of E2 (1 nM to 1 μM) tested at 12 and 24 h confirmed that E2 did not induce the expression of EZH2 in U251 cells. These results contrast with those reported by Bhan et al. in breast cancer cell lines, whose EZH2 expression was induced by E2 (22). Although GBMs and breast cancer are estrogen-responsive tumors, they have different biological contexts in which the role of EZH2 and the mechanisms controlling its expression may be different, as has been suggested in neoplastic cells (14). EZH2 acts as an oncogene in various neoplasms such as breast cancer, prostate cancer, and GBMs (19, 38, 39). However, it can act as a tumor suppressor in lymphomas and ovarian cancer (40–42). These data provide evidence of the complexity of the mechanisms regulating EZH2 expression and that its activity depends on the cellular context.

As mentioned earlier, E2 induces the proliferation, migration, and invasion of cell lines derived from human GBM (4, 8, 9); these processes are also promoted by EZH2 (13, 19, 21) activity. Therefore, we evaluated the impact of EZH2 silencing and EZH2 inhibition on these E2-induced processes. Since U251 presented the highest levels of EZH2, we decided to focus on it for subsequent analyses. E2 failed to induce proliferation, migration, and invasion when EZH2 was silenced or inhibited pharmacologically in U251 cells, suggesting that EZH2 mediates E2 effects on GBM cell lines. Although it has been previously reported that inhibition of EZH2 suppressed GBM growth, migration, and reversed EMT *in vitro* and *in vivo* (24, 43–45), this study is the first description demonstrating that EZH2 mediates E2 pro-oncogenic actions on GBM cells. Our results contribute to understanding the molecular mechanisms underlying GBM progression induced by E2 and EZH2 epigenetic mechanisms’ participation.

The above observations indicated that EZH2 mediates E2-induced pro-oncogenic actions in GBM cells. Since E2 acts through ERα, and ERβ, it raises the possibility of a relation between EZH2 and ERs in GBMs. Using transcriptomic data of LGG, GBM, and NT, we verified the previous evidence that EZH2 was overexpressed in glioma and correlated to the degree of tumor progression (19–21). An earlier report of our group described a high ERβ expression in glioma and low ERα expression compared to NT (9). At first glance, these data suggest that in gliomas, ERs and EZH2 expressions are not related. Nevertheless, we decided to evaluate this possibility in detail in GBM samples.

GBM is a highly heterogeneous tumor among gliomas at both the molecular and cellular levels (46). According to transcriptomic data, EZH2 mRNA was highly and homogeneously expressed in GBM biopsies, supporting its relevant role in GBM tumors. On the other hand, ERα and ERβ expressions were highly heterogeneous. This characteristic allowed us to define three types of sample clusters and compare their mRNA expression profiles. In terms of the expression of ERs, especially ERα, and terms of the number of differentially expressed genes, cluster 3 (high ERα and ERβ) exhibit more differences concerning cluster 2 (relatively low ERα and medium ERβ) than cluster 1 (high ERα and very low ERβ). It is worth mentioning that within each of these hierarchical subgroups, the sex ratio is maintained, so sex is not related to the levels of expression of the ERs according to which the three hierarchical groups were defined. When analyzing the expression of the set of early estrogenic response genes, these were enriched in GBM cluster 3 compared to the other two clusters. In contrast, just as EZH2 expression was similar among GBM clusters, the expression of the target genes of PRC2, the regulatory complex to which EZH2 belongs, was also homogeneous. These results show that high expression of ERs in GBM cluster 3 is associated with increased modulation of gene expression and reveal the leading role of ERα in such actions, including E2 target genes in GBM samples.

These results reveal a complex and heterogeneous mechanism underlying ERs actions in GBM. In this context, an essential role of ERα in GBM progression has been indicated by the observations of E2 induced cell growth of astrocytoma cell lines (4) and EMT activation in human GBM-derived cells through ERα (9). While, reports indicated that E2 increased proliferation from GBM (4), treatment with various ERβ agonists reduced GBM cell proliferation (47). In other words, these data show that different ER subtypes modulate different actions in GBM. Also, we must consider that ERs expression levels may not always be proportional to their activity (48). In breast cancer cells, ERβ significantly modifies a subset of ERα-dependent splicing (49). So, the cumulative sum of the particular actions of different ERs at a specific cellular context will define the overall cancer cell phenotype.

We performed a GO analysis to get an overall picture of the gene functions differentially modulated in GBM cluster 3 (high ERα and ERβ) regarding the other GBM clusters with distinct ERs expression. Among biological/functional pathways mainly upregulated in GBM cluster 3, several are associated with pro-oncogenic actions promoted by E2 in cancer, even some of which are already described in GBM. For example, crosstalk between matrix components and ERs contributes to ECM remodeling and EMT in breast cancer (23). In GBM cluster 3, ECM organization/disassembly and cell-matrix adhesion terms were highly upregulated. Estrogens also regulate the expression of genes that affect vesicle trafficking, including exocytosis, which affects the growth and metastasis of breast cancer cells (50, 51). Gene term of exocytosis regulated was overexpressed in GBM cluster 3. Increased expression of genes associated with platelet degranulation in GBM cluster 3 was also observed. Platelets play an essential role in cancer as they release permeability factors, degradative enzymes, and growth and angiogenic factors that assist tumor development progression and metastasis. In the ERs-positive subgroup of breast tumors, a high abundance of proteins related to platelet degranulation has been described (52). Integrin-mediated signaling pathway was also upregulated in cluster 3. It is known that ERβ promotes migration through the Integrin β1/MMP-9 pathway in normal colon epithelial cells (53). Importantly, outlining these upregulated gene terms, it emerges that most of them are compatible with the increase of migration and invasion processes induced by E2 in GBM cells (4, 8, 9).

Intriguing data concerning the downregulation of genes related to processing mRNA splicing, regulation of G2/M transition in cell cycle, and CBs associated process in GBM cluster 3 were found. It has been reported that ERβ significantly affects ERα induced mRNA splicing in estrogen-responsive breast cancer cells (50), suggesting that ERβ has considerably different and, in most cases, opposite biological effects compared to ERα. Regarding G2/M process modulation by E2, in human cervical cancer cells, non-classical membrane estrogen receptors G protein-coupled receptor (GPER) activation induced G2/M cell cycle arrest *via* EGFR/ERK1/2 signals (54). However, the participation of ERα and ERβ in this sense is unknown. CBs mediate small nuclear and nucleolar ribonucleoproteins and telomerase assembly and modification. CBs are enriched in transcriptionally active and or with high splicing demands, such as cancer cells (55). Our analysis indicates significant deregulation of several pathways associated with CBs in GBM cluster 3 (high ERα and ERβ).

The accumulated evidence of non-genomic signaling initiated by steroid receptors at membrane occurs in various cell types (10, 56). E2 can induce dimerization of ERs at the plasma membrane inducing fast signals by second messengers (e.g., cAMP, cGMP, Ca2+) and or activates kinase cascades that, in turn, may modulate nuclear transcription factors functions (57). In this regard, it has been reported that E2 regulates the activity of EZH2 through non-genomic signaling mediated by ERα and ERβ leads, which leads to phosphorylation of EZH2 by AKT and MAPK pathways in benign and cancer prostate cells (36). Furthermore, post-translational modification of EZH2 can regulate its activity. For example, Akt phosphorylates EZH2 at serine 21 and suppresses its methyltransferase activity by impeding EZH2 binding to histone H3, which results in the derepression of silenced genes (58). Specifically, in GBM stem cells, AKT phosphorylates EZH2, and then it methylates STAT3 leading to enhanced STAT3 activity, which promotes GSC self-renewal and tumor malignancy (39). Recent work reported that overexpression of ERβ isoform 5 (ERβ5) induced AKT phosphorylation and activation of STAT3, besides promoting migration of GBM cell lines (59). Persistent activation of STAT3 could be crucial for tumor progression and epithelium-mesenchyme transition (59, 60), a process also regulated by E2 in GBMs (9). Interestingly, in the set of evaluated biopsies, GBM samples have the highest expression of ERβ, opening the possibility of assessing its participation in an extra-nuclear mechanism of E2 for mediating EZH2 activity in GBM cells.

In summary, in this work, we showed that in GBM cells, E2 induced proliferation, migration, and invasion through EZH2 without modulating its expression. Furthermore, in GBM samples, the expression levels of EZH2 are not related to those of the ERs, nor those of their target genes, suggesting that E2 induces EZH2 activation through a non-genomic mechanism that needs to be studied to clarify the outline of E2 pro-oncogenic activities in GBM.

## Data Availability Statement

The datasets analyzed in this study can be found in: https://portal.gdc.cancer.gov/ and https://gtexportal.org/home/.

## Author Contributions

AM-M, JO, AH-V, KH-O, and KP-G carried out the experiments, data collection, and analysis. AM-M and IC-A conceived the experiments. AM-M and KH-O wrote the first draft of the manuscript, and IC-A contributed to the revision and editing of the manuscript. All authors have read and agreed to the published version of the manuscript.

## Funding

This work was financially supported by Programa de Apoyo a Proyectos de Investigación e Innovación Tecnológica (PAPIIT), project number: IN217120. AD-M received a fellowship funding from CONACYT (CVU894530).

## Conflict of Interest

The authors declare that the research was conducted in the absence of any commercial or financial relationships that could be construed as a potential conflict of interest.

## Publisher’s Note

All claims expressed in this article are solely those of the authors and do not necessarily represent those of their affiliated organizations, or those of the publisher, the editors and the reviewers. Any product that may be evaluated in this article, or claim that may be made by its manufacturer, is not guaranteed or endorsed by the publisher.

## Abbreviation

BrdU, 5-Bromo-2’deoxyuridine; CBs, Cajal bodies; E2, 17β-estradiol; ECM, extracellular matrix; EED, 1 embryonic ectoderm development; EMT, epithelial to mesenchymal transition; EREs, estrogen response elements; ERs, estrogen receptors; ERα, estrogen receptors α; ERβ, estrogen receptors β; EZH2, Zeste 2 Enhancer Homologous enzyme; GBM, glioblastoma; GO, gene ontology; GPER, G protein-coupled receptor; GSEA, gene set enrichment analysis; H3K27me1/2/3, histone 3 lysine 27 mono, di, and trimethylation; PcG, Polycomb Group; PCR1, Polycomb 1 repressor complex; PRC2, Polycomb repressor complexes; SET, Su(var) 3-9; E(z) Trithorax domain; SUZ12, Suppressor of Zeste 12; TCGA, The Cancer Genome Atlas; TSS, transcription start site; WD40, tryptophan–aspartate repeat motifs.
