# Supplementary material for: EZH2 Mediates Proliferation, Migration, and Invasion Promoted by Estradiol in Human Glioblastoma Cells
Source: Front Endocrinol (Lausanne). 2022 Feb 7;13:703733. doi: 10.3389/fendo.2022.703733 (PMC8859835; doi:10.3389/fendo.2022.703733)
Supplement: Supplementary file 1 [file DataSheet_1.docx]

Supplementary Material

**EZH2 mediates proliferation, migration, and invasion promoted by estradiol in human glioblastoma cells**

**Aylin Del Moral-Morales, Juan Carlos González-Orozco, Ana María Hernández-Vega, Karina Hernández-Ortega, Karla Mariana Peña-Gutiérrez and Ignacio Camacho-Arroyo^*^**

***Correspondence:** Dr. Ignacio Camacho-Arroyo: [camachoarroyo@gmail.com](mailto:camachoarroyo@gmail.com)


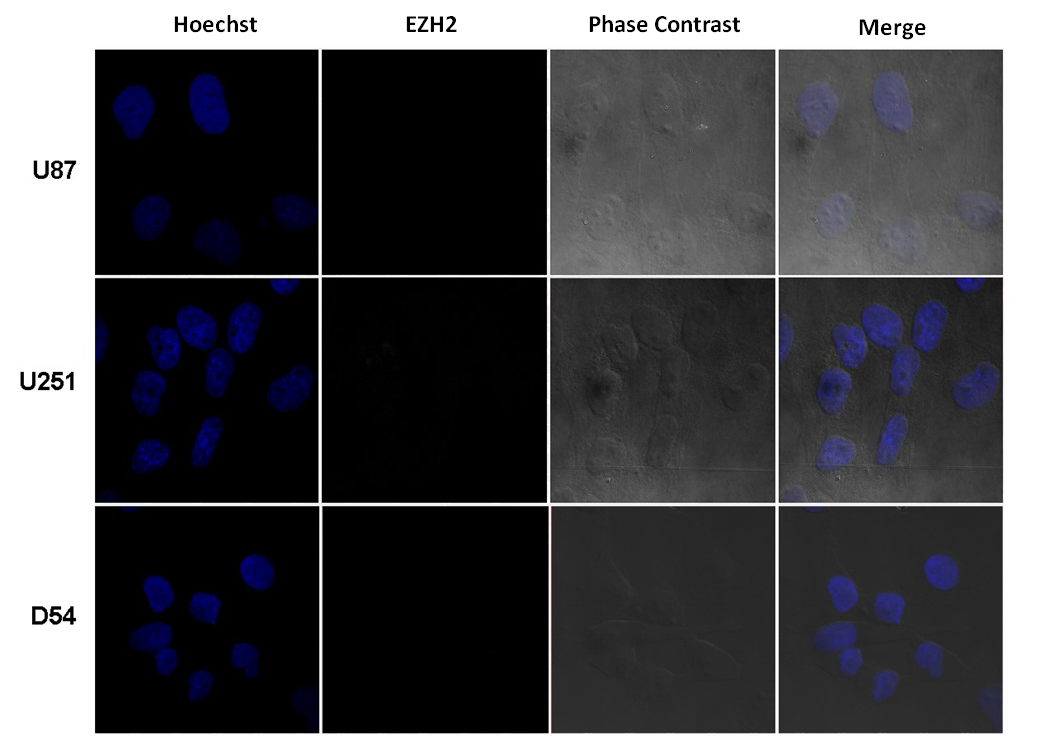


**Supplementary Figure 1.** Negative control of immunofluorescence for EZH2 in GBM cells. Representative images of immunofluorescence assays performed without the primary antibody against EZH2. No signal was detected in the channel corresponding to EZH2; only nuclei marked with the Hoechst dye are distinguished.


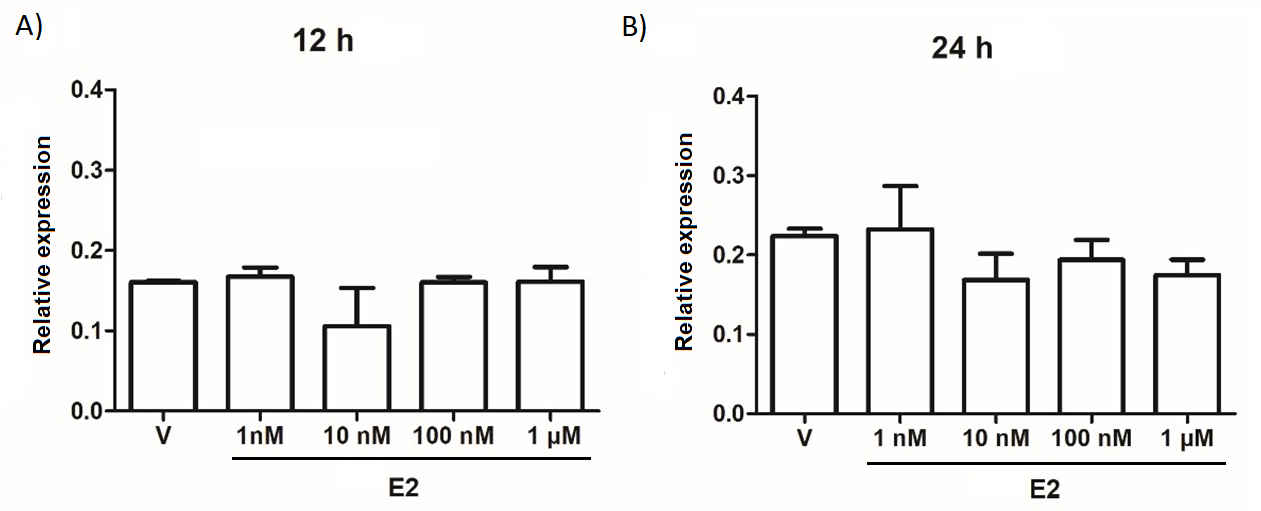


**Supplementary Figure 2.** Effect of E2 on EZH2 expression in U251 cell line. EZH2 mRNA expression was quantified by RT-qPCR in cells treated with E2 (1 nM, 10 nM, 100 nM and 1 μM) or V (CDX 0.02%) for **A**) 12 and **B**) 24 hours. Graphs show EZH2 relative expression (normalized to 18S ribosomal RNA by 2^∆∆Ct^ method). Each bar represents the mean ± S.E.M. *n = 3*.


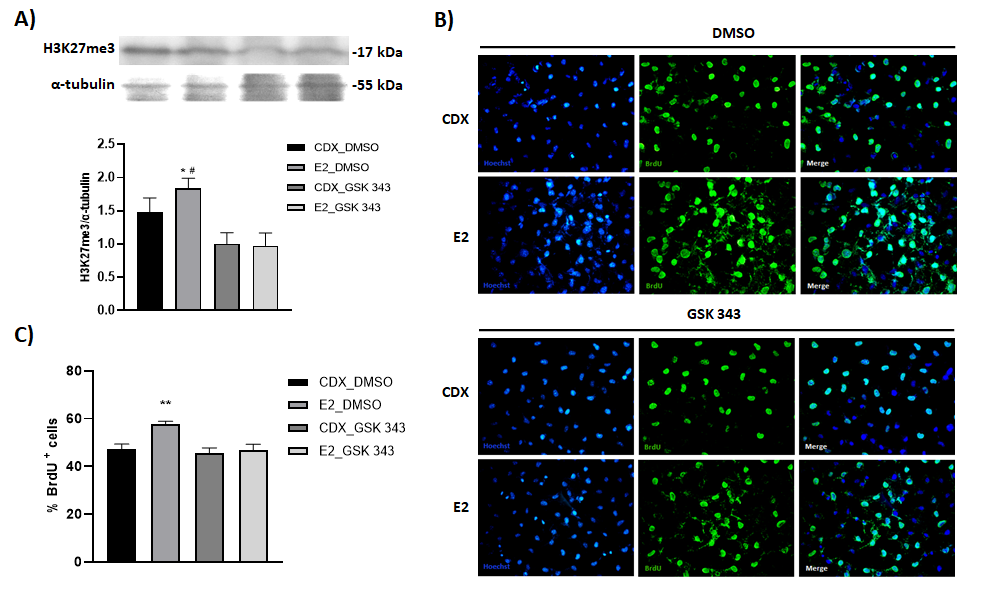


**Supplementary Figure 3**. GSK343 treatment downregulates H3K27me3 and decreases proliferation in U251 cells treated with E2. (**A**) Levels of H3K27me3 in U251 cells treated with CDX, E2 (10 nM) and E2 (10 nM) + GSK343 (5 μM) for 24h time were determined by western blot analysis. α-tubulin was used as a loading control. Bars represent the mean ± S.E.M. n =3. * p<0.05 vs CDX_GSK 343, # p<0.05 vs E2_GSK 343. (**B**) Representative images of BrdU incorporation in proliferating U251 cells, (**C**) and its corresponding quantification graphs showing the percentage of BrdU positive cells. Bars represent the mean ± S.E.M. n =3. **p<0.01 vs the other groups.


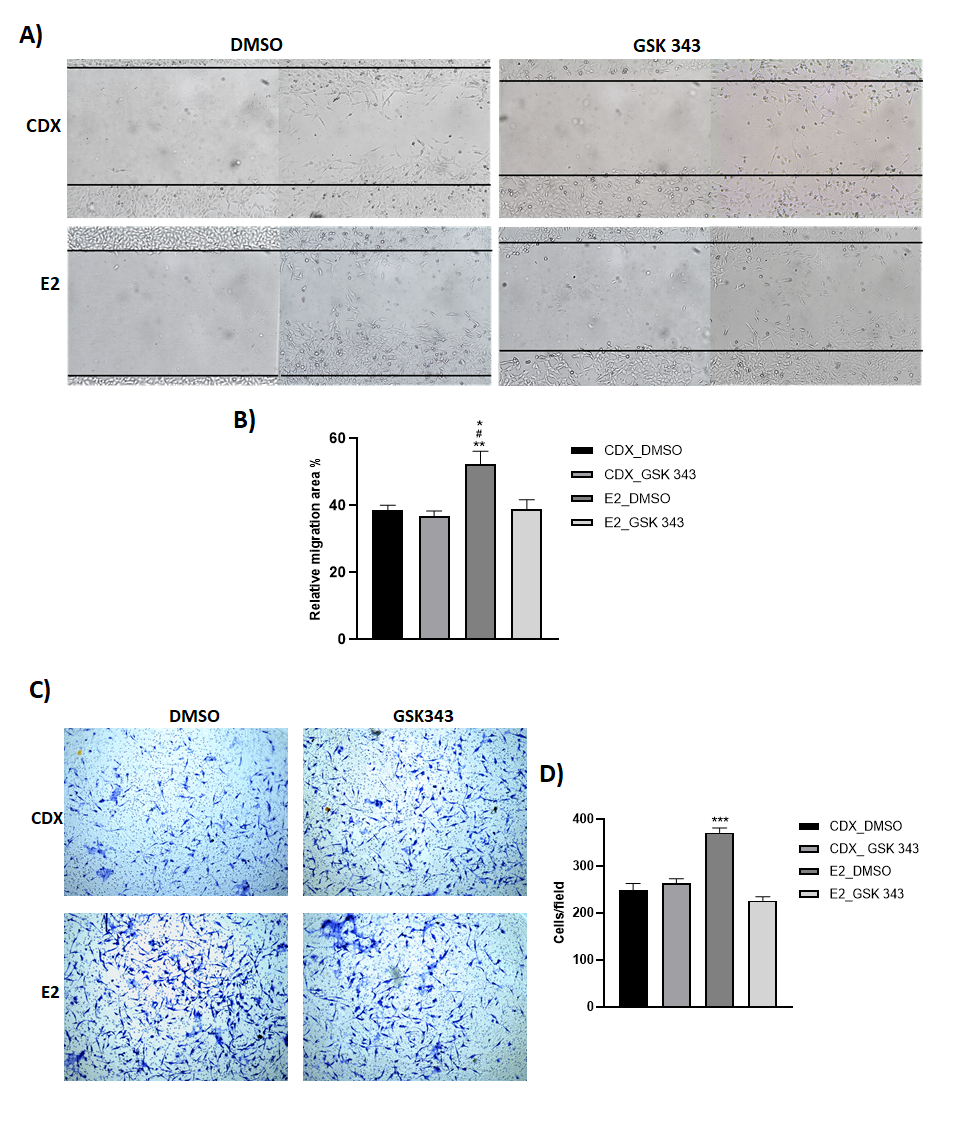


**Supplementary Figure 4**. Effect of GSK 343 on E2-induced migration and invasion. (A) Wound healing assays were performed in the U251 cell line treated with the EZH2 inhibitor, GSK 343 or vehicle DMSO (0.01%) plus E2 (10 nM), or CDX (0.02 %). Representative images of the scratch area at 0 and 24 h. (B) Relative migration area (%) of U251 cells into wound area was measured 24 h after scratch formation. Graph data are shown as mean ± S.E.M., n = 3. *p<0.05 vs CDX_DMSO, ** p<0.01 vs CDX_GSK343, #p <0.05 vs E2_GSK343. (C) Transwell assays were carried out in U251 cells treated as previously described. Representative images of invading cells at the bottom of the transwells (cells that penetrated matrigel) stained with cresyl violet=0.1 % were captured 24 h after treatments. The total number of invading cells by field was quantified. (D). Results are expressed as the mean ± S.E.M., n = 3. ***p <0.001 vs the other groups.

**
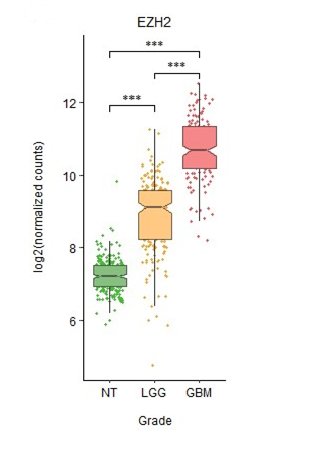
**

**Supplementary Figure 5.** EZH2 expression in human glioma biopsies and healthy brain cortex samples. Ribonucleic acid sequencing (RNA-seq) counts of low-grade gliomas (LGG, n=167) and GBM (n=155) obtained from The Cancer Genome Atlas TCGA, and expression profiles of 249 healthy brain cortex samples (normal tissue, NT) obtained from the GTEx database were normalized. Differential expression analysis of EZH2 is shown in graphs. ***p<0.001.


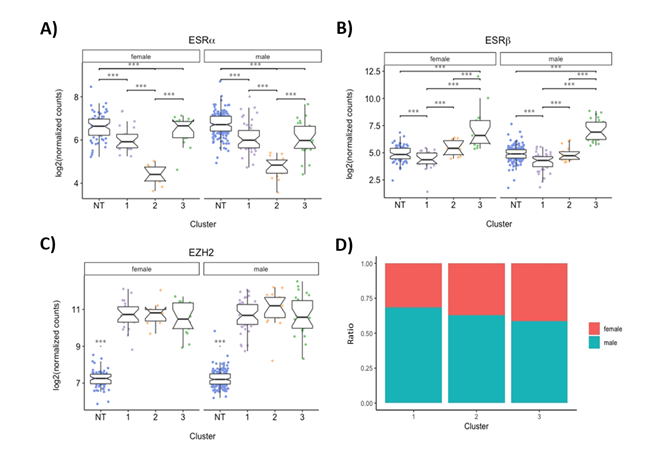


**Supplementary Figure 6.** Supplementary Figure 6. Sex-segregated expression of ERα, ERβ, and EZH2 in clusters of human glioma biopsies and healthy brain cortex samples. Expression profiles of 139 primary GBM obtained from TCGA and RNA seq data of 249 healthy brain cortex samples (normal tissue, NT) obtained from the GTEx database were analyzed. (A) ERα, (B) Erβ, and (C) EZH2 expression was analyzed by sex in the hierarchical clusters of GB groups and NT. (D) The sex ratio is maintained in all three GB clusters. (Chi-squared test = 1.0393, df = 2, p-value = 0.5947).


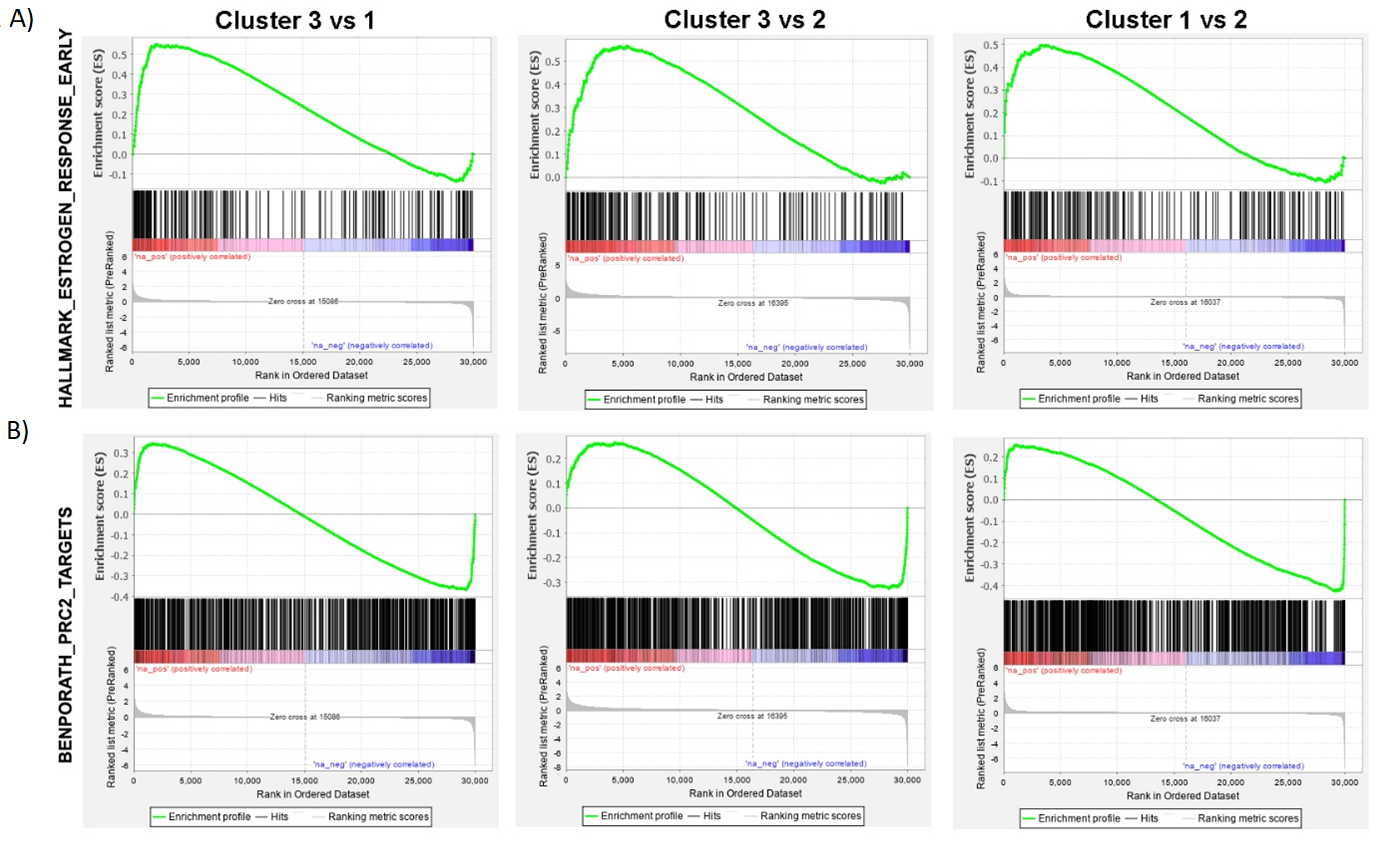


**Supplementary Figure 7.** Gene Set Enrichment Analysis (GSEA) of transcriptomic data of GBM samples obtained from TCGA. GSEA enrichment plots of differential expression of gene sets related to A) the early estrogenic response and B) proven targets of the PRC2 complex in GBM sample clusters. A higher enrichment of early estrogen-responsive genes in group 3 than in groups 1 and 2 was observed, based on their normalized enrichment score (ES) and normalized P-value. In plots, genes are sorted by signal/noise ratio according to their differential expression between each pair of GBM sample clusters compared. Vertical bars indicate genes in each gene set, and the ES is depicted in green.

| **Supplementary Table 1**. Cell viability of U251 human GBM cells treated with E2 and EZH2 siRNA | |
| --- | --- |
| **Treatment** | **% Cell viability** |
| CDX_control siRNA | **82.6 ± 2.43** NS |
| CDX_EZH2 siRNA | **80.9 ± 2.97** NS |
| E2_control siRNA | **83.4 ± 3.62** NS |
| E2_ EZH2 siRNA | **80.8 ± 4.99** NS |
|  |  |
| All values are expressed as mean ± SEM. Data were analyzed by one-way ANOVA followed by a Tukey´s multiple comparison test. NS= Non significant. | |

**Supplementary Table 1**. Effect of EZH2 silencing on viability U251 cells. The table summarizes the percentage of cell viability obtained by Trypan blue exclusion assay after 24 h of E2 (10nM) or vehicle (CDX, 0.02%) treatments. Results are presented as the mean ± S.E.M., n = 3. Data were analyzed by one-way ANOVA followed by Tukey´s multiple comparison test. NS = Non significant.
